# N-(9-Acridinyl) Amino Acid Derivatives: Synthesis and In Vitro Evaluation of Anti-*Toxoplasma gondii* Activity

**DOI:** 10.3390/pharmaceutics17030374

**Published:** 2025-03-15

**Authors:** Đorđe Zlatković, Vladimir Dobričić, Jelena Srbljanović, Olivera Lijeskić, Neda Bauman, Vladimir Ćirković, Tijana Štajner

**Affiliations:** 1 National Reference Laboratory for Toxoplasmosis, Group for Microbiology and Parasitology, Center of Excellence for Food- and Vector-born Zoonosis, Institute for Medical Research, National Institute of Republic of Serbia, University of Belgrade, 11129 Belgrade, Serbia; djordje.zlatkovic@imi.bg.ac.rs (Đ.Z.); jelena.srbljanovic@imi.bg.ac.rs (J.S.); olivera.lijeskic@imi.bg.ac.rs (O.L.); neda.bauman@imi.bg.ac.rs (N.B.); vladimir.cirkovic@imi.bg.ac.rs (V.Ć.); 2 Department of Pharmaceutical Chemistry, University of Belgrade-Faculty of Pharmacy, Vojvode Stepe 450, 11000 Belgrade, Serbia; vladimir.dobricic@pharmacy.bg.ac.rs

**Keywords:** synthesis, N-(9-acridinyl) amino acid derivatives, *Toxoplasma gondii*, cytotoxicity

## Abstract

**Background/Objectives**: Acridine, an aromatic heterocyclic compound, serves as a basis for the synthesis of potent bioactive derivatives, displaying a broad spectrum of biological activity, such as antibacterial, antitumor, and antiparasitic activity. With the ability to undergo various types of electrophilic substitutions, introducing different side chains could lead to compounds being active towards various and potentially multiple biotargets. *Toxoplasma gondii*, a ubiquitous protozoan parasite with worldwide distribution, poses a major health threat, particularly in immunocompromised patients and fetuses. Current treatment options for toxoplasmosis are scarce, with notable limitations, especially regarding side myelotoxicity and inactivity towards *T. gondii* cysts, causing a need for novel drug candidates. The aim of this study was to evaluate selected N-(9-acrydinil) amino acid derivatives as potential anti-*T. gondii* agents. **Methods**: Synthesis of new derivatives was performed using a two-step method, with the initial mixing of 9-chloroacridine with methanol and sodium alkoxide solution and subsequent adding of appropriate amino acids. Cytotoxicity of the tested compounds was evaluated on the Vero cell line using a MTT assay, while their anti-*T. gondii* activity was investigated using *T. gondii* RH strain tachyzoites. **Results**: CC_50_ values of the derivatives ranged from 41.72 to 154.10 µM. Anti-*T. gondii* activity, displayed as a reduction in the number of viable tachyzoites compared to the untreated control, ranged from 0 to 33.3%. One of the derivatives displayed activity comparable to the standard treatment option while retaining acceptable cytotoxicity. Esterification, presence of aromatic substituents and the length of the amino acid side chain were identified as key factors that affect both toxicity and activity of these derivatives. **Conclusions**: Promising results obtained throughout this study provide guidelines for further structural modifications of N-(9-acrydinil) amino acid derivatives in order to synthesize drug candidates competitive to standard treatment options for toxoplasmosis.

## 1. Introduction

Acridine is a heterocyclic compound with a planar aromatic structure that exhibits interesting chemical properties due to its conjugated system and a nitrogen atom in the pyridine ring. Amongst others, acridine has the ability to undergo various types of electrophilic substitution reactions, which makes it an interesting choice for derivatization that could lead to compounds active towards various biotargets [1]. Acridine derivatives have attracted much attention due to their broad spectrum of biological activity, such as antibacterial, antitumor, anti-Alzheimer, anti-inflammatory and antiparasitic activity. Amsacrine is an acridine derivative that is still in use as an antitumor agent in some countries, while in others it is withdrawn from use due to serious toxicity. However, recent studies of antitumor activity and toxicity towards healthy cells suggest that with proper modification of acridine’s side chain (e.g., introduction of amino acids at the C9 position of acridine ring), new antitumor derivatives with minimal toxicity could be obtained [2]. Although different modes of action seem to be exerted by acridines depending on their therapeutic targets, their most consensual mechanism of action is interaction with the DNA [3]. The cytochrome *bc_1_* complex is a frequent drug target against Apicomplexa, not only in malaria-inducing *Plasmodium* sp., but also in *Toxoplasma gondii*, the causative agent of toxoplasmosis [4].

*T. gondii* is a ubiquitous protozoan parasite with a worldwide distribution that infects up to one third of the human population [5]. In a joint WHO/FAO report on the significance of parasites transmitted via food, *T. gondii* was ranked fourth and toxoplasmosis was declared a disease of global concern and as number one in Europe [6,7]. Furthermore, *T. gondii* is a very significant opportunistic infectious agent in immunocompromised patients, not only in HIV-infected patients, but also in patients treated by both hematopoietic stem cell transplantation (HSCT) and solid organ (heart, liver, kidney, etc.) transplantation procedures, who are vulnerable and increasing in number [8].

The life cycle of *T. gondii* is complex and involves three life stages. Sporozoites are excreted within oocysts in the environment, through the feces of an infected cat (animal of the Felidae family), a definite host. In intermediate hosts, including humans, the parasite exists in the form of tachyzoites and bradyzoites. Tachyzoites are a vegetative, rapidly dividing and disseminating form, responsible not only for parasitaemia in the acute stage of acquired infection, but also for the manifestations of reactivation of chronic infection. In immunocompetent hosts, tachyzoites convert into quiescent bradyzoites that remain encysted within nucleated cells (tissue cysts), predominantly in predilection tissues—CNS including retina, cardiac and striate muscles, for extended periods of time, even life-long, resulting in chronic toxoplasmosis. Human infection is usually acquired by ingesting parasites in contaminated food or water and is asymptomatic in over 80% of immunocompetent individuals. Tissue cysts induce a potent and protective immunity, but even the most competent immune response fails to eradicate the cysts. Moreover, immunosuppression of chronically infected hosts triggers the reactivation of toxoplasmosis, rupture of tissue cysts, egress of bradyzoites, and their re-conversion to proliferating tachyzoites, which then disseminate causing life-threatening (focal and/or systemic) infection [9].

Current treatment options for toxoplasmosis are scarce and have limited potential in immunosuppressed patients due to their side toxicity. The main target of anti-*Toxoplasma* drugs is the folate pathway, involved in DNA synthesis with the dihydrofolate reductase (DHFR) and dihydropteroate synthetase (DHPS) enzymes. Two main treatment options, pyrimethamine (PYR) and trimethoprim (TMP), both act on parasite DHFR, but are unable to distinguish it from the enzymes of the human host. They are not efficient enough as monotreatment, hence they are usually administered in combination regimens with sulfonamides, most often sulfadiazine (SDZ) or sulfamethoxazole (SMX), which inhibit DHPS. A combination of PYR+SDZ is the first-choice treatment in 80% of cases [10,11]. Even though PYR and SDZ are inhibitors of DNA synthesis in *T. gondii* tachyzoites, they may also inhibit DNA synthesis in tissues with high metabolic activity, such as the bone marrow and epithelium. Along with side effects, mainly myelotoxicity, the therapeutic choice can be guided by the availability or price of drugs. Indeed, PYR is unavailable and/or unaffordable in many countries, thus explaining the frequent use of the less efficient combination of TMP+SMX as the first line treatment [12,13]. Furthermore, none of the available treatment options are by any means efficient towards *T. gondii* cysts, solely acting on circulating tachyzoites.

Therefore, it is of great importance to identify novel potent drug candidates that would be well-tolerated in vulnerable categories of patients and would act on both tachyzoites and cysts. Our aim was to explore the anti-*T. gondii* potential of selected (previously and newly synthesized) N-(9-acrydinil) amino acid derivatives (AAD), and to identify structural elements of AADs with the highest influence on activity. Even though certain promising results were recently published on acridones (derivatives somewhat similar to AADs), this is the first study focused on evidence-based, experimental evaluation of the anti-*T. gondii* activity of AADs [14].

## 2. Materials and Methods

### 2.1. Materials

#### 2.1.1. Chemicals Used for Synthesis, Purification and Physiochemical Characterization

Chloroform (stabilized with amylene, ≥99%), acetonitrile (for analysis, ≥99.5%) and methanol (extra dry, for synthesis) were obtained from Fisher Scientific (Loughborough, UK). 9-Chloroacridine (97%), 6-aminohexanoic acid, 4-aminobutyric, β-alanine, L-phenylalanine, sodium pieces and diethyl ether (≥99.7%) were purchased from Sigma Aldrich (Steinheim, Germany). Deuterated dimethyl sulfoxide (used in NMR characterization) and 8-aminooctanoic acid were purchased from Acros Organics (Geel, Belgium). Silica gel 60 GF_254_ for preparative thin layer chromatography was obtained from Merck (Darmstadt, Germany).

#### 2.1.2. Cell Culture

The Vero cell line (ATCC^®^ CCL-81), derived from the kidney tissue of the Green African monkey (*Chlorocebus sabaeus*), was utilized as a model of non-infected cells in the cytotoxicity assay. The cells were thawed from stocks maintained in liquid nitrogen at the National Reference Laboratory (NRL) for Toxoplasmosis and cultured in D10 medium—Advanced Dulbecco’s Modified Eagle Medium (DMEM) (Thermo Fisher Scientific, Waltham, MA, USA), supplemented with 10% bovine calf serum (BCS) (Sigma-Aldrich, St. Louis, MO, USA), 1% HEPES buffer (Thermo Fisher Scientific, Waltham, MA, USA), and 1% penicillin-streptomycin (Pen/Strep) solution (Thermo Fisher Scientific, Waltham, MA, USA) in T25 and T75 flasks. Depending on confluency, cells were subcultured 1–2 times per week.

#### 2.1.3. Parasites

*T. gondii* RH strain tachyzoites were obtained from the NRL for Toxoplasmosis, Institute for Medical Research, Belgrade, Serbia, where the RH strain is regularly maintained via passages using female Swiss-Webster mice twice a week (Ethical license no. 323-07-02445/2014-05/1).

### 2.2. Methods

#### 2.2.1. N-(9-Acrydinil) Amino Acid Derivatives Synthesis

Out of 10 selected AADs in total, five (AAD1–5) were previously screened for antitumor activity [2]. In addition, five more derivatives (AAD6–10) were newly synthesized using a modified procedure based on previously published methodology, omitting the isolation of the expected 9-alkoxy intermediate and adopting a one-pot synthesis approach [2,15].

The first step of synthesis consisted of mixing 9-chloroacridine (0.3 mmol, 1 eq.) with methanol (3 mL) and a freshly prepared sodium alkoxide solution (a solution of sodium methoxide in methanol, 3 mL). This was followed by two and a half hours of refluxing of the reaction mixture. The second step of synthesis was adding the appropriate amino acid (0.42 mmol, 1.4 eq.) to the reaction mixture with an additional four-hour refluxing. The reaction mixture was then evaporated to dryness. Purification was carried out using preparative thin-layer chromatography.

**Scheme 1:**
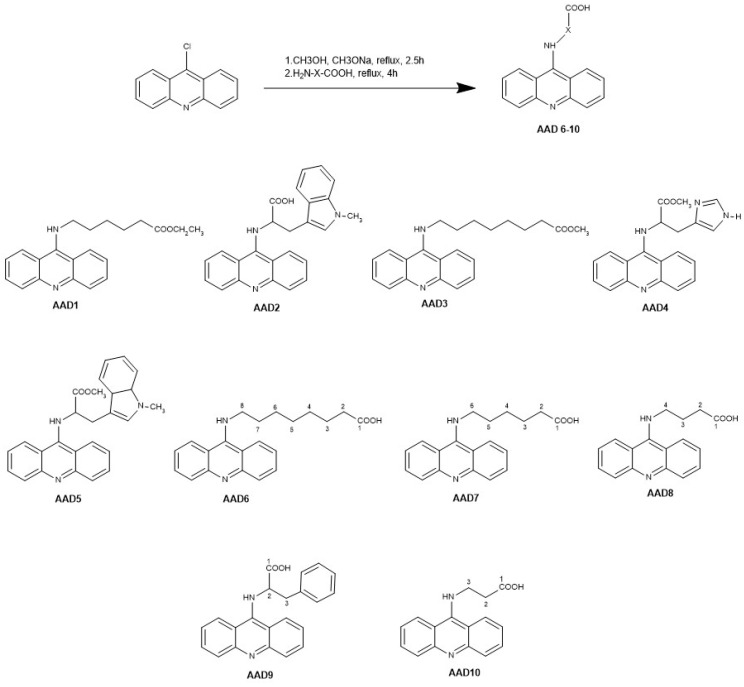
Synthesis and chemical structure of all tested AADs; AAD—N-(9-acrydinil) amino acid derivative.

The procedure of synthesis and chemical structures of all tested AADs are presented in Scheme 1.

#### 2.2.2. Structural Characterization of Derivatives

Structural characterization of newly synthesized derivatives (Scheme 1, AAD6-10) was performed by measuring melting points and employing spectroscopic methods such as IR, NMR, MS/MS, and HRMS. The Boetius PHMK 05 (Radebeul, Germany) was used for melting point determination, while a Nicolet iS10 spectrometer (Thermo Fisher Scientific Inc., Madison, WI, USA) was used for IR spectra analysis. NMR analyses were performed using a BRUKER AVANCE III 400 NMR spectrometer (Bruker Biospin GmbH, Rheinstetten, Germany). Finally, the TSQ Quantum Access MAX mass spectrometer (Thermo Fisher Scientific Inc., Madison, WI, USA) was used for obtaining MS/MS data, and accurate mass measurements were conducted with an Orbitrap Exploris 240 UHPLC-HRMS system (Thermo Fisher Scientific Inc., Madison, WI, USA). For purity testing, HPLC analysis was performed on an Agilent 1200 chromatograph (Agilent Technologies, Palo Alto, CA, USA), then equipped with a binary pump, manual injector (20 µL sample loop) and DAD detector. The column used was Zorbax Extend C18, acquired from Agilent Technologies, Palo Alto, CA, USA (150 mm × 4.6 mm, 5 µm particle size), while the mobile phase was a mixture of acetonitrile and water (pH was adjusted to 3.2 using phosphoric acid) in the following ratios (*v*/*v*): 30:70 (AAD6, AAD7 and AAD9) and 20:80 (AAD8 and AAD10). Column temperature was adjusted to 25°C, while the flow rate was 1 µL/min. UV detection was performed at 220, 230, 254, 265 and 280 nm. For AAD9, an additional purity testing method (method B) was employed (at the same chromatograph), including the chiral stationary phase, to evaluate the enantiomeric purity of the mentioned derivative (mobile phase: mixture of acetonitrile and 10 mM amonium acetate at ratio (*v*/*v*) of 15:85; column: Chiralpak^®^ HSA (Daicel Chiral Technologies, Illkirch-Graffenstaden, France); column temperature: 25°C; flow rate: 0.9 µL/min; UV detection wavelengths: 220, 230, 254, 265 and 280 nm). Purity testing results, ranging from 96.2% to 98.5% for previously synthesized AADs (AAD1-5), were obtained using a similar method, then described and published by Rupar et al. [2].

**8-(acridin-9-ylamino) octanoic acid (AAD6).** This derivative was synthesized according to the previously described procedure using sodium methoxide solution and 8-aminooctanoic acid. The reaction mixture was purified using preparative thin layer chromatography with mobile phase chloroform/methanol 9:2 (*v*/*v*). A yellow crystalline solid was obtained after recrystallization in diethyl ether. Yield: 30%. Purity: 99.1%. Mp 149.7–151.2 °C. IR (ATR) ν = 750.092, 1532.132, 1566.505, 1588.554, 1633.468, 1633.69, 3345.478 cm^−1^. ^1^H NMR (400 MHz, DMSO-*d*_6_) δ ppm 7.35–8.43 (m, 8 H, Ar-H), 3.91 (t, 2 H, *J* = 7.2 MHz, 2 × H-8), 2.14 (t, 2 H, *J* = 7.2 MHz, 2 × H-2), 1.78 (m, 2 H, 2 × H-7), 1.44 (m, 2 H, 2 × H-3), 1.21–1.34 (m, 6 H, 2 × H-4, 2 × H-5, 2 × H-6). ^13^C NMR (100 MHz, DMSO-*d*_6_) δ 175.19, 154.62, 132.50, 125.83, 122.71, 50.03, 45.86, 34.25, 30.42, 28.91, 28.86, 26.69, 24.90. *m*/*z* = 337.2 (M^+^+1), 195.14, 194.14, 207.14, 178.15, 169.15, 319.18. HRMS [M+H]^+^ calculated for C22H27O2N2 = 337.19105; observed = 337.18978.

**6-(acridin-9-ylamino) hexanoic acid (AAD7).** This derivative was synthesized according to the previously described procedure using sodium ethoxide solution and 6-aminohexanoic acid. The reaction mixture was purified using preparative thin layer chromatography with mobile phase chloroform/methanol 9:2 (*v*/*v*). A yellow crystalline solid was obtained after recrystallization in diethyl ether. Yield: 30%. Purity: 95.5%. Mp 210.0–212.0 °C. IR (ATR) ν = 748.936, 1168.038, 1472.070, 1531.364, 1567.867, 2944.858 cm^−1^. ^1^H NMR (400 MHz, DMSO-*d*_6_) δ ppm 7.37–8.50 (m, 8 H, Ar-H), 3.95 (t, 2 H, *J* = 7 MHz, 2 × H-6), 2.17 (m, 2 H, 2 × H-2), 1.83 (m, 2 H, 2 × H-5), 1.35–1.54 (m, 4 H, 2 × H-3 and 2 × H-4). ^13^C NMR (100 MHz, DMSO-*d*_6_) δ 175.00, 155.36, 144.40, 143.11, 133.15, 126.07, 122.88, 121.89, 114.56, 110.51, 49.67, 34.15, 29.92, 26.37, 24.66. *m*/*z* = 309.2 (M^+^+1), 195.19, 177.98, 268.33, 206.88, 184.08, 225.12. HRMS [M+H]^+^ calculated for C21H25O2N2 = 309.15975; observed = 309.15880.

**4-(acridin-9-ylamino) butanoic acid (AAD8)**. This derivative was synthesized according to the previously described procedure using sodium methoxide solution and 4-aminobutyric acid. The reaction mixture was purified using preparative thin layer chromatography with mobile phase chloroform/methanol 9:2 (*v*/*v*). A yellow crystalline solid was obtained after recrystallization in diethyl ether. Yield: 41%. Purity: 97.1%. Mp 231.3–232.6 °C. IR (ATR) ν = 658.285, 743.686, 1466.726, 1533.507, 1588.35, 1564.915, 3316.456 cm^−1^. ^1^H NMR (400 MHz, DMSO-*d*_6_) δ ppm 7.33–8.47 (m, 8 H, Ar-H), 3.98 (m, 2 H, 2 × H-4), 2.38 (m, 2 H, 2 × H-2), 2.00 (m, 2 H, 2 × H-3). ^13^C NMR (100 MHz, DMSO-*d*_6_), δ ppm 175.95, 142.92, 133.12, 131.22, 130.05, 127.42, 126.35, 124.10, 122.77, 121.41, 114.79, 50.18, 33.16, 25.82. *m*/*z* = 281.2 (M^+^+1), 195.17, 263.17, 87.35, 193.17, 207.17, 206.17. HRMS [M+H]^+^ calculated for C19H21O2N2 = 281.12845; observed = 281.12766.

**(S) 2-(acridin-9-ylamino)-3-phenylpropanoic acid (AAD9)**. This derivative was synthesized according to the previously described procedure using sodium methoxide solution and L-phenyalanine. This derivative was purified using preparative thin layer chromatography with mobile phase chloroform/methanol 9:2 (*v*/*v*). A yellow crystalline solid was obtained after recrystallization in diethyl ether. Yield 14%. Purity: method A—95.7%, method B—97.4%. Mp 166.1–167.9 °C. IR (ATR) ν = 705.0, 699.323, 745.834, 1471.696, 1531.565, 1566.386, 1587.161 cm^−1^. ^1^H NMR (400 MHz, DMSO-*d*_6_) δ ppm 7.37–8.33 (m, 8 H, Ar-H (acridine ring)), 7.00–7.08 (m, 5 H, Ar-H (phenyl ring)), 4.95 (s, 1 H, H-2). ^13^C NMR (100 MHz, DMSO-*d*_6_) δ 141.38, 138.11, 133.87, 129.83, 129.75, 129.25, 128.93, 128.79, 128.41, 126.71, 126.44, 121.43, 120.91, 117.82, 63.91, 38.23. *m*/*z* = 343.1 (M^+^+1), 240.22, 302.0, 195.19, 307.31. HRMS [M+H]^+^ calculated for C23H20N2O2 = 343.14410; observed = 343.14297.

**3-(acridin-9-ylamino) propanoic acid (AAD10).** This derivative was synthesized according to the previously described procedure using sodium methoxide solution and β-alanine. The reaction mixture was purified using preparative thin layer chromatography with mobile phase chloroform/ethanol 9:3 (*v*/*v*). A yellow crystalline solid was obtained after recrystallization in diethyl ether. Yield: 20%. Purity: 98.4%. Mp 207.0–208.5 °C. IR (ATR) ν = 658.854, 740.644, 1427.941, 1536.810, 1557.430, 3395.767 cm^−1^. ^1^H NMR (400 MHz, DMSO-*d*_6_) δ ppm 7.34–8.41 (m, 8 H, Ar-H), 4.17 (t, 2 H, *J* = 6.4 MHz, H-3), 2.69 (t, 2 H, *J* = 6.4 MHz, H-12). ^13^C NMR (100 MHz, DMSO-*d*_6_) δ 133.55, 126.35, 122.82, 120.52, 46.48, 46.47, 41.45, 40.10, 35.08, 29.23. *m*/*z* = 267.2 (M^+^+1), 207.19, 206.19, 179.22, 193.18, 194.20, 221.16. HRMS [M+H]^+^ calculated for C18H19O2N2 = 267.11280; observed = 267.11195.

#### 2.2.3. Solubility and Solution Stability of the Synthesized Derivatives

Solubility and stability testing was conducted for three of the derivatives, evaluated as potential in vivo testing candidates, namely AAD1, AAD2 and AAD8.

Solubility was evaluated using a visual inspection method, checking the clarity and opalescence of different concentrations (up to 30 mM) of dimethyl sulfoxide (DMSO) solutions of the mentioned AADs, as well as of their further dilutions with the pH 7.4 phosphate buffer (100-, 1000- and 10,000-times dilutions).

The stability of the synthesized AADs was evaluated in mediums with three different pH values: 2 (0.01 M HCl), 5.5 (phosphate buffer) and 7.4 (phosphate buffer). Stability was tested by employing a HPLC method, using an Agilent 1200 chromatograph (Agilent Technologies, Palo Alto, CA, USA) and Zorbax Extend C18 column (Agilent Technologies, Palo Alto, CA, USA). The mobile phase consisted of acetonitrile and water (pH was adjusted to 3.2 using phosphoric acid) at a ratio of 30:70 (*v*/*v*) for all three AADs. Column temperature was adjusted to 25 °C, while the flow rate was 1 µL/min. UV detection was performed at 220, 230, 254, 265 and 280 nm. In short, samples (solutions of the investigated AADs in the appropriate mediums) were injected twice into the HPLC column: the first time right after the preparation of the solutions, and the second time after the stability testing interval—two hours for the pH 2 solution and four hours for the pH 5.5 and 7.4 solutions. At the end of the incubation intervals, the decrease in peak areas and occurrence of additional peaks on chromatograms were analyzed in comparison with the chromatograms obtained right after the preparation of the solutions.

#### 2.2.4. Cytotoxicity Assay

The MTT colorimetric assay, a widely used method for assessing the cytotoxicity of newly synthesized compounds, was used to evaluate the toxic effect of AADs on non-infected cells. This assay is based on the ability of metabolically active, viable cells to reduce the yellow 3-(4,5-dimethylthiazol-2-yl)-2,5-diphenyltetrazolium bromide (MTT) reagent to purple formazan. The number of viable cells in the well is directly proportional to the intensity of the resulting color. The MTT assay was performed according to the manufacturer’s instructions using the In Vitro Toxicology Assay Kit (MTT-based, Sigma-Aldrich, St. Louis, MO, USA), with a protocol specifically modified to accommodate the cell line and derivatives analyzed in this study [2,16].

In brief, Vero cells were seeded in a 96-well plate, at a density of 25,000 cells per well and incubated for 24 h at 37 °C in a 5% CO_2_ atmosphere. After the incubation period, the supernatant was removed from each well and replaced with serial dilutions of the analyzed derivatives, as well as with the combination of PYR+SDZ (Sigma-Aldrich, St. Louis, MO, USA) as a positive control. All compounds were investigated in five different concentrations, ranging from 100 μM to 6.25 μM, using a two-fold serial dilution method. The positive control was tested in the range of 0.4 μM + 100 μM to 0.025 μM + 6.25 μM (ratio of PYR+SDZ adjusted according to the literature [17]). In negative control wells, containing only non-infected Vero cells, the discarded supernatant was replaced by a new amount of D10 medium. Blank wells, used to account for any background signal from the colored culture medium, contained only D10 medium. All samples, including controls and blanks, were analyzed in triplicates.

Samples containing Vero cells with added AADs or the positive control were incubated for 72 h. After incubation, the supernatant was discarded, and a new amount of D10 medium (100 μL) was added to each well. The MTT reagent (10 μL, 5 mg/µL) was subsequently added to each well, and the plates were incubated for an additional four hours. After the incubation period, the supernatant was discarded, and the resulting formazan crystals were dissolved using the solubilizing solution from the In Vitro Toxicology Assay Kit. Absorbance was measured at 540 nm using an ELISA plate reader (Multiskan EX, Thermofisher Scientific, Waltham, MA, USA). Data analysis was conducted, and CC_50_ values (cytotoxic concentration used to lyse/inhibit average of 50% of cells) were obtained using nonlinear regression in GraphPad Prism 10.4.1 (GraphPad Software Inc., La Jolla, CA, USA).

#### 2.2.5. Anti-*T. gondii* Activity Screening

The anti-*T. gondii* activity of the AADs was evaluated according to previously described methodology, slightly modified by using the DMEM culture medium supplemented with 10% bovine calf serum (BCS) without antibiotics (referred to as modified D10 medium) [18]. RH strain *T. gondii* tachyzoites were harvested from the intraperitoneal exudate of infected mice during routine passage of the strain at the NRL for toxoplasmosis. The sample (intraperitoneal lavage fluid of RH strain-infected mice) was then washed three times using Dulbecco Phosphate Buffered Saline (DPBS) (Thermofisher Scientific, Waltham, MA, USA) and centrifuged at 2.500 rpm for five minutes to obtain a pellet consisting of RH strain tachyzoites only. The pellet was then resuspended, and the parasite count was determined by Trypan blue vital staining, using a Bürker-Türk counting chamber. Tachyzoites were resuspended in an appropriate volume of DMEM culture medium to achieve the required tachyzoite concentration. A suspension of tachyzoites (2 × 10^6^ tachyzoites/well, in 100 μL volume) was then seeded into a 96-well microplate and exposed to a 25 µM concentration of 10 AADs, as well as a combination of PYR+SDZ, for 24 h. All samples, including the untreated control consisting of RH strain tachyzoites in a modified D10 culture medium, were tested in triplicates.

After 24 h, 20 µL of sample was collected from each well, mixed with an equal volume of Trypan blue stain, and the number of viable tachyzoites was determined using a Bürker-Türk counting chamber. The reduction in the number of viable tachyzoites compared to untreated control was calculated using the following formula:

(1) $$\mathrm{Reduction}\;\%=100\%-(\frac{\mathrm{median}\;\mathrm{value}\;\mathrm{of}\;\mathrm{number}\;\mathrm{of}\;\mathrm{viable}\;\mathrm{tachyzoites}\;\mathrm{in}\;\mathrm{test}\;\mathrm{wells}}{\mathrm{median}\;\mathrm{value}\;\mathrm{of}\;\mathrm{number}\;\mathrm{of}\;\mathrm{viable}\;\mathrm{tachyzoites}\;\mathrm{in}\;\mathrm{control}\;\mathrm{wells}}\times 100\%)$$

#### 2.2.6. Molecular Docking Analysis

Crystal structures of selected *T. gondii* targets were retrieved from the PDB databank—PRP4K kinase (pdb code: 7Q4A), enoyl acyl carrier protein reductase (pdb code: 2O2S), ornithine aminotransferase (pdb code: 5E5I), cystathionine gamma-lyase (pdb code: 8BIS), calcium-dependent protein kinase 1 (pdb code: 4WG3), thymidylate synthase-dihydrofolate reductase (pdb code: 4KYA) and prolyl-tRNA synthetase (pdb code: 5XIG). The receptor sites for molecular docking were created using MAKE Receptor 3.2.0.2, according to the coordinates of ligands co-crystalized with these targets [19]. Prior to the molecular docking, ligand preparation was performed in OMEGA 2.5.1.4 and files containing conformers for each ligand were generated. The OEDocking 3.2.0.2, which employs the FRED (fast exhaustive docking) tool, was used for the analysis of ligand binding poses in the receptor sites [20,21,22]. Exhaustive scoring was performed using the Chemgauss4 scoring function. Further optimization was performed using OEChemscore scoring function. Scoring and consensus pose selection were performed using the Chemgauss4 scoring function. Other settings were set as default.

## 3. Results

### 3.1. Cytotoxicity

All 10 AADs were evaluated for their cytotoxic effects on non-infected cells using a MTT assay. Derivatives with previously established antitumor activity (AAD1-5) displayed CC_50_ values ranging from 41.72 to 133.70 µM. These results were used as the basis for synthesis of other derivatives. Hence, AAD6-10 displayed CC_50_ values ranging from 50.19 to 154.10 µM. CC_50_ values of all 10 AADs and the PYR+SDZ combination are shown in Table 1.

**Figure 1:**
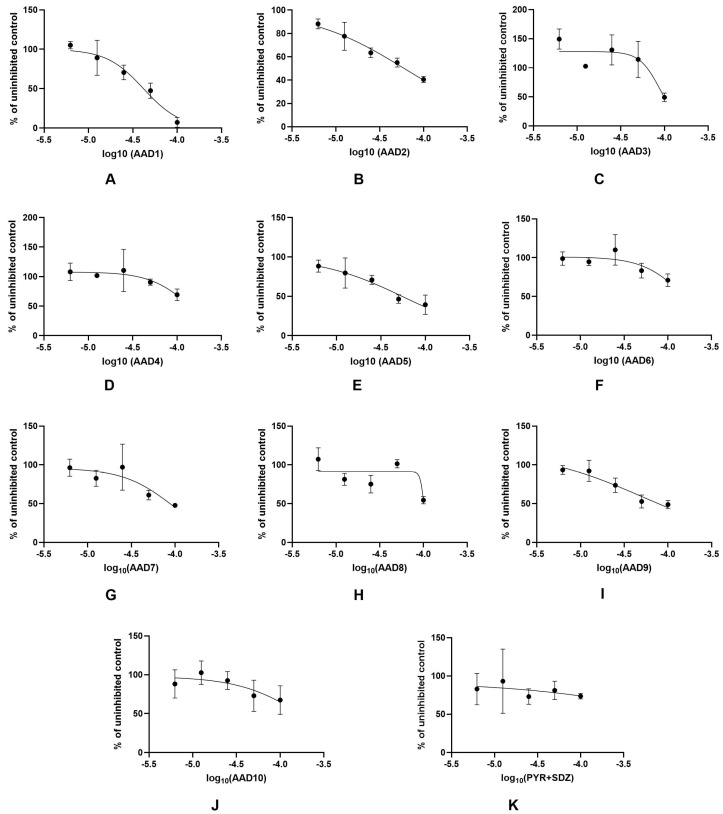
Dose response cytotoxicity curves for analyzed AADs: (**A**) AAD1; (**B**) AAD2; (**C**) AAD3; (**D**) AAD4; (**E**) AAD5; (**F**) AAD6; (**G**) AAD7; (**H**) AAD8; (**I**) AAD9; (**J**) AAD10; and of the control drug combination (**K**) PYR+SDZ.; AAD—N-(9-acrydinil) amino acid derivatives; PYR+SDZ—pyrimethamine and sulfadiazine combination treatment.

Dose response cytotoxicity curves, generated in GraphPad Prism 10.4.1. for all 10 investigated AADs, as well as for the control treatment (PYR+SDZ combination), are presented in Figure 1.

### 3.2. Anti-T. gondii Activity

As in cytotoxicity testing, antiparasitic activity was determined for previously synthesized derivatives (AAD1-5) first, with a subsequent impact of obtained results on the design of other newly synthesized derivatives. Acridine derivatives with an antitumor effect (AAD1-5) displayed a reduction in the number of viable tachyzoites ranging from 4.4% (AAD4) to 25.5% (AAD2), while the reduction rates for other newly synthesized derivatives (AAD6-10) ranged from 20.9% (AAD7) to 33.3% (AAD8). Anti-T. gondii activity of all 10 AADs compared to the untreated control and PYR+SDZ combination is presented in Table 2.

### 3.3. In Silico Analysis of Interactions with Potential Targets

Docking (FRED Chemgauss4) scores of all tested compounds are presented in Table 3. Each column represents molecular docking results for one target, and column titles are the corresponding pdb codes.

A more negative (lower) score represents stronger binding to a target, so AADs with similar or lower scores than the corresponding co-crystallized ligands were underlined. All AADs showed higher docking scores than co-crystallized ligands when docked to calcium-dependent protein kinase 1 (4WG3) and thymidylate synthase-dihydrofolate reductase (4KYA), and their interactions with these targets were not further analyzed. In addition, the only AAD that showed a similar docking score for ornithine aminotransferase (5E5I) was AAD4, but it did not show any important interactions with the target in comparison to the co-crystallized ligand, so this target was also not taken into consideration.

**Figure 2:**
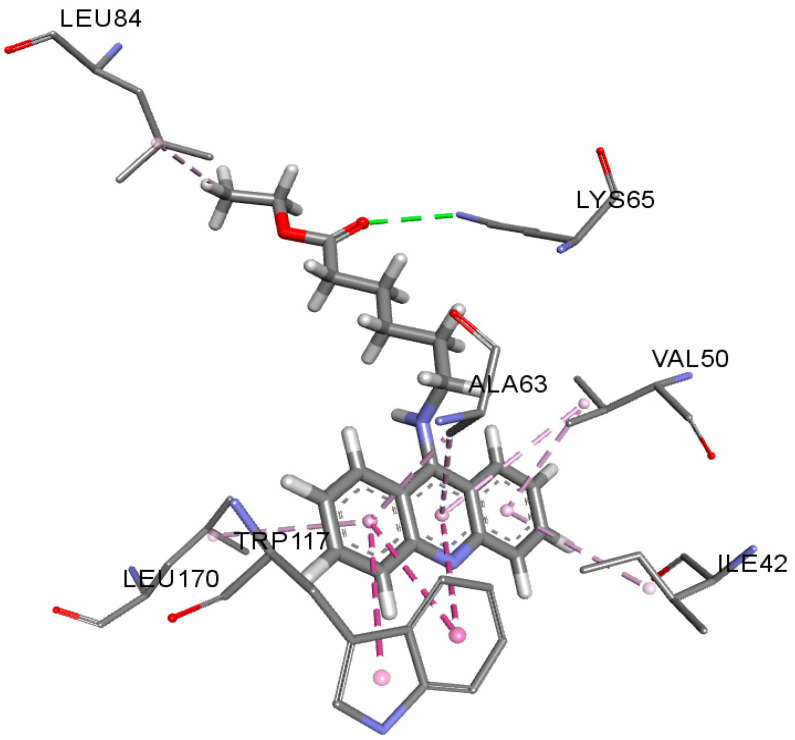
Interactions of AAD1 with PRP4K kinase.

The best (lowest) docking scores for PRP4K kinase (7Q4A) were calculated for AAD1, AAD3 and AAD5, which were similar to the co-crystallized ligand (altiratinib). AAD1 also showed the most similar interactions in comparison to altiratinib (hydrogen bond with LYS65, as well as π-π, π-σ and π-alkyl interactions with ALA63, TRP117, LEU170, LEU84, PHE115 and PHE183 (Figure 2).

**Figure 3:**
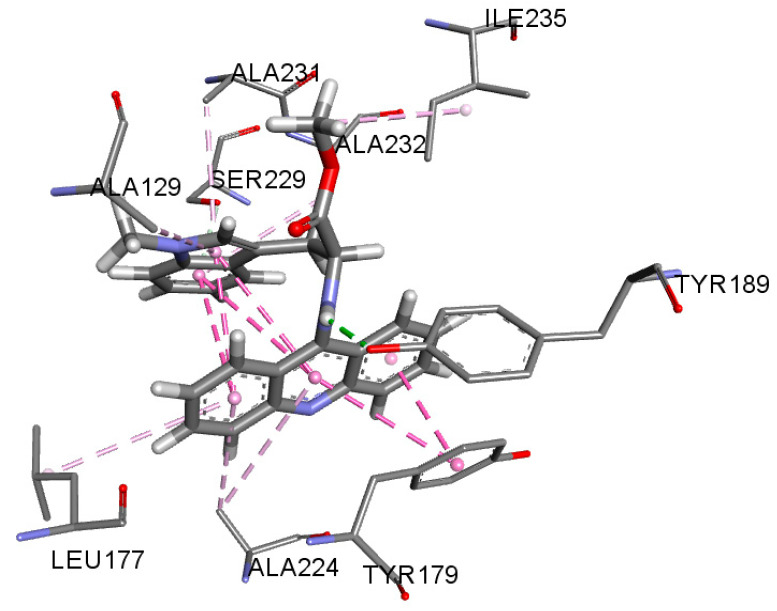
Interactions of AAD5 with enoyl acyl carrier protein reductase.

The best (lowest) docking scores for enoyl acyl carrier protein reductase (2O2S) were calculated for AAD2, AAD5 and AAD9, which were similar to the co-crystallized ligand (triclosan). All these acridines showed similar interactions in comparison to triclosan (π-π, π-σ and π-alkyl interactions with ALA231, ILE235, TYR179, and MET193). In contrast to triclosan, these acridines form an additional hydrogen bond with TYR189, which probably strengthens interaction with the enzyme (Figure 3).

**Figure 4:**
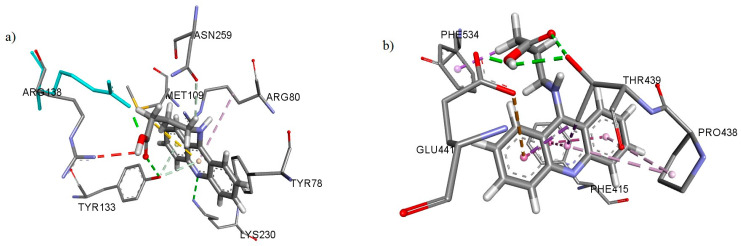
Interactions of AAD8 with (**a**) cystathionine gamma-lyase and (**b**) prolyl-tRNA synthetase.

The best docking score for cystathionine gamma-lyase (8BIS) was calculated for AAD8, which was lower compared to the co-crystallized ligand (DL-propargylglycine). This acridine also showed similar interactions in comparison to the co-crystallized ligand (hydrogen bonds with TYR133 and LYS230, Figure 4a).

The best docking scores for prolyl-tRNA synthetase (5XIG) were calculated for AAD2, AAD7 and AAD8, which were similar to the co-crystallized ligand (a quinazolin-4-one derivative). All these acridines showed similar interactions in comparison to the co-crystallized ligand (π-π, π-σ and π-alkyl interactions with PRO438, PHE534 and PHE415, as well as a hydrogen bond with GLU441, Figure 4b).

## 4. Discussion

Acridine, or in fact N-(9-acridinyl) amino acid derivatives, may have already been considered as a possible anti-*T. gondii* agent due to promising results reflecting its activity on another Apicomplexan parasite, *Plasmodium* sp., the causative agent of malaria, which is the most prevalent parasitic disease worldwide [23,24,25]. Their antitumor activity could further add to the interest, since immunosuppressed patients undergoing cancer treatment are at significant risk of either acquired or reactivated toxoplasmosis, one of the major opportunistic infections [2]. However, this is the first study focused on an evidence-based, experimental evaluation of the anti-*T. gondii* activity of N-(9-acridinyl) amino acid derivatives.

Due to the structural similarity between *Toxoplasma* sp. and *Plasmodium* sp., and literature data on the promising antimalarial activity of several groups of acridine derivatives, we have chosen our previously synthesized AADs as a starting point for this experimental setting [23,24,25]. Both cytotoxicity and anti-*T. gondii* activity testing of previously synthesized derivatives (AAD1-5) were conducted prior to the synthesis of AAD6-10, and the obtained results were used as a starting point for their design. The aim of the design and synthesis presented in this paper was to expand the set of previously synthesized AADs by introducing more derivatives, with both aliphatic and aromatic side chains. Since only AAD1 and AAD3 contained aliphatic side chains, we opted to synthesize derivatives with the same length of the side chain but with free COOH group (AAD7 and AAD6), as well as derivatives with shorter aliphatic side chains (AAD8 and AAD10), to cover the range of 3–8 carbon atoms in the side chain. Even though three of the previously synthesized derivatives had an aromatic side chain, we opted to design and synthesize another aromatic side chain derivative (AAD9) with a free COOH group, since a similar derivative (AAD2) displayed the highest activity among previously synthesized derivatives. Since the AAD2 derivative, containing an unesterified amino acid in its structure, displayed lower cytotoxicity (higher CC_50_) and drastically better anti-*T. gondii* activity compared to its ester form (AAD5), we opted for all newly synthesized AADs (AAD6-10) to be derivatives of unesterified amino acids. Future experiments will include design and synthesis of AADs with longer amino acid aliphatic side chains, branched aliphatic side chains, polyfunctional amino acids, side chains with some common anti-*T. gondii* pharmacophores, as well as their analogues with the same side chain but a substituted acridine ring.

Synthesis of the AAD6-10 was conducted according to the procedure employed in the synthesis of AAD1-5, previously described by Rupar et al. [2]. Newly synthesized derivatives were obtained in yields ranging from 14 to 41%. The extremely low yield of AAD9 (14%) was due to the presence of side products of the synthesis that could be observed on TLC plate, which aggravated its purification process.

Results indicating the cytotoxicity of non-infected cells obtained for all 10 derivatives analyzed in this study align with the previously published data, with CC_50_ values falling into micromolar range [2]. In general, derivatives of unesterified amino acids of all the investigated pairs of acid/ester compounds (AAD2 and AAD5, AAD6 and AAD3, AAD7 and AAD1) displayed higher CC_50_ values (Table 1) and were proven to be less toxic than the ester analogues. According to the cytotoxicity curves (Figure 1), an increase in the concentration of AADs resulted in increased cell toxicity; hence, the cytotoxic effect of these compounds is dose dependent. Dose dependency is generally a favorable property of newly synthesized compounds, since it implies that the difference between effective and toxic concentrations could potentially be established.

In spite of the initial data based on the evaluation of AAD2 effects vs. AAD5 that indicated lower toxicity and higher anti-*T. gondii* efficacy of derivatives of unesterified amino acids, which were further elaborated by data obtained for AAD6 (the first of the newly synthesized AADs, derivative of unesterified amino acid of the AAD3), this advantage did not hold for all derivatives of unesterified amino acids (AAD6-10). Moreover, the evaluation of AAD7, a derivative of the unesterified amino acid of AAD1, reflected slight deterioration in activity, despite favorable CC_50_ values (Table 1 and Table 2). The contradiction of these findings demonstrates a need for further and broader research, including more of the similar ester-/unesterified amino acid pairs of AADs, in order to clarify the impact of this part of the structure on biological activity.

The results obtained throughout this study further imply that medium length side chains (four and six carbon atoms) present in AAD8 and AAD1 correlate with higher activity compared to derivatives containing longer side chains (eight carbon atoms, AAD3 and AAD6) or shorter side chains (three carbon atoms, AAD10). The correlation between the length of the aliphatic side chain and the activity of the derivative was also established in previous studies, in which the longer side chain derivatives displayed lower antiproliferative activity [2,15]. These findings strongly indicate that the choice of amino acid used for derivatization of the acridine ring has crucial effect on the toxicity and activity of synthesized compounds.

On the other hand, the introduction of the aromatic substructure in the amino acid side chain yielded incoherent results, with AAD2 displaying higher activity, AAD9 displaying medium activity and AAD5 displaying no activity (Table 2). The presence of an aromatic ring in the amino acid side chain also led to, on average, lower CC_50_ values (higher toxicity) of these compounds compared to compounds containing aliphatic side chains (Table 1).

Overall, AAD8, a derivative of unesterified amino acids with a medium length side aliphatic chain, displayed both the highest reduction percentage (33.3%) of the number of viable *T. gondii* tachyzoites, closely matching the antitoxoplasmic activity of PYR+SDZ (Table 2), and acceptable cytotoxicity (CC_50_ = 102 µM) (Table 1). Still, the PYR+SDZ combination, a standard treatment option, displayed the lowest cytotoxicity and the highest anti-*T. gondii* activity, highlighting the need for further modifications of AADs. Structural modifications such as the introduction of pharmacophores that inhibit specific targets on *T. gondii* or modification of the acridine ring by addition of substituents with appropriate electronic and steric properties could result in drug candidates superior to current treatment options. The introduction of substituents with electron-donating properties in the acridine ring may result in increased bioactivity, since these substitutions lead to an increase in electron density, as well as lead to the changes in interactions crucial for DNA binding and intercalation ability of the AADs [14,26]. Additionally, introducing the hydroxamate-based pharmacophores, preferably in the side chain part of the molecule, should lead to histone deacetylase (HDAC) inhibition, which was shown to result in potent anti-*T. gondii* activity of some experimental compounds [27,28]. Furthermore, certain acridine derivatives displayed the ability to inhibit protein kinase systems, and since *T. gondii* calcium-dependent protein kinase 1 (TgCDPK1) is a known drug target, introduction of the substituents recognized to provide TgCDPK1 inhibition and selectivity, such as 6-alkoxy-2-naphthyl and 4-piperidinylmethylene groups, could potentially broaden the mechanism of action and improve the overall activity of the AADs tested in this research [29,30].

As of late, it was shown that acridones, another group of compounds which also incorporate the acridine ring as a core pharmacophore, exhibited substantially higher in vitro activity towards *T. gondii* compared to the AADs. So, the results reported by Alday et al. on acridone derivatives provide a distinct perspective [14]. The presence of a keto group at the C9 position in acridone molecule appears to significantly enhance their bioactivity, with these compounds demonstrating picomolar efficacy in an in vitro model of acute toxoplasmosis. This boosted activity is likely attributable to their ability to inhibit cytochrome *bc*_1_ and other ubiquinone-binding enzymes. Furthermore, the anti-*T. gondii* activity of acridone derivatives correlated with their antimalarial efficacy, as compounds with the most potent antimalarial effects also exhibited the highest in vitro efficacy in the experimental model of acute toxoplasmosis. These findings suggest that incorporating established antimalarial pharmacophores, particularly those targeting cytochrome *bc_1_*, into AADs could yield compounds with significantly improved activity [31]. However, the potential impact of such modifications on cytotoxicity remains to be determined, since the selectivity towards the parasite’s mitochondrial electron transport chain constituents can often be a problem when designing novel experimental compounds.

Furthermore, established correlation between antimalarial and anti-*T. gondii* activity could also explain the initially observed difference in activity between derivatives of unesterified amino acids and ester forms of AADs. Previous research demonstrated that an increase in lipophilicity, which would occur with the esterification of the derivatives of unesterified amino acids form of the AADs, led to lower antimalarial activity, which potentially could also translate into lower anti-*T. gondii* activity [32]. The same research implied that the acidity of the synthesized compounds could also influence activity, since the derivatives of unesterified amino acids would be fully ionized at physiological pH levels, thus being less lipophilic. Additionally, a substitution in the acridone ring outside of the pyridine subunit was determined to significantly increase activity in contrast to the unsubstituted aromatic scaffold as present in AADs evaluated in our research [14].

Of particular interest is the antiproliferative activity of the AADs, especially activity reported on the K562 cancer cell line (chronic myelogenous leukaemia), which could certainly be interesting from the aspect of designing dual activity agents with both efficacy on tumor cells (due to their effect on DNA and topoisomerase) and *T. gondii* [2]. Since hematological malignancies are the main indication for HSCT, and patients undergoing HSCT are at a high risk of reactivated toxoplasmosis, synthesis of compounds that could be further developed towards a unique treatment option that would at the same time restrain toxoplasmosis and tumor cells, should allow for a reduction of immunosuppressant cytotoxic drugs in the HSCT protocol [8]. Bypassing the myelotoxicity of standard options used for prophylaxis (TMP+SMX) and treatment (PYR+SDZ) of toxoplasmosis would not only improve the outcome of HSCT, but also improve the survival rate of these patients [8,10]. The preliminary requirement for these vulnerable categories of patients should be embodied in a drug candidate of acceptable toxicity and high efficacy towards tachyzoites, inhibiting or diminishing them as soon as they enter circulation, with an antitumor effect as an incredibly precious bonus. However, even then, the relapses in chronically infected immunosuppressed patients could not be avoided without a treatment option active on *T. gondii* bradyzoites difficult to reach in intracellular cysts. Hence, evaluation of activity towards *T. gondii* bradyzoites is a logical next step for AADs investigated in this study, as it is for other newly synthetized compounds efficient on tachyzoites.

Three of the investigated derivatives displaying the most potent anti-*T. gondii* activity, which were tested for solubility and stability, proved to be adequately soluble in DMSO, making clear and transparent solutions up to 30 mM, while maintaining solubility when being diluted up to 10,000 times in a pH 7.4 phosphate buffer. Stability testing showed no additional chromatographic peak appearances and no significant reduction in peak area after the incubation periods for all three tested AADs in all three tested solutions, making these derivatives stable in set conditions. These results indicate that these three AADs (AAD1, AAD2 and AAD8) will be good candidates for systemic administration and in vivo studies of toxicity and efficacy.

In order to identify potential mechanisms of action of compounds AAD1-AAD10, molecular docking was performed. A thorough analysis of a protein data bank revealed that several crystal structures of *T. gondii* enzymes (in complex with corresponding inhibitors) are available: PRP4K kinase, enoyl acyl carrier protein reductase, ornithine aminotransferase, cystathionine gamma-lyase, calcium-dependent protein kinase 1, thymidylate synthase-dihydrofolate reductase and prolyl-tRNA synthetase. For each target, docking scores of all tested AADs were calculated, and then only for the best ranked molecules, a deeper analysis of interactions with the enzyme was performed, and these interactions were compared to those of the corresponding inhibitor. A tested compound is considered a potential enzyme inhibitor if the docking score is similar or better (lower), and if the interactions are similar in comparison to the corresponding inhibitor.

Molecular docking results showed that the following enzymes could be potential targets of some of the acridines tested in this study: PRP4K kinase (plays a role in regulating transcription and the spindle assembly checkpoint), enoyl acyl carrier protein reductase (enzyme involved in fatty acid synthesis), cystathionine gamma-lyase (breaks down cystathionine into cysteine, 2-oxobutanoate (α-ketobutyrate), and ammonia) and prolyl-tRNA synthetase (involved in protein synthesis) [33,34,35,36]. Therefore, our *in silico* study revealed that tested AADs could interfere with *T. gondii* protein and lipid synthesis.

The most common mechanism of action of compounds with the acridine ring is DNA intercalation. Although the crystal structure of the *T. gondii* DNA was not available in the protein data bank and molecular docking could not be performed on this target, it can be expected that DNA intercalation and the subsequent obstruction of its replication can also be expected from tested AADs.

Although providing promising results, this study is limited by a rather small number of derivatives tested for anti-*T. gondii* activity in vitro by using only one strain, which could imply that these results are not merely due to structural characteristics of AADs but are also *T. gondii*-strain-dependent. However, this is the first study indicating anti-*T. gondii* activity of AADs with antitumor potential, pointing out directions for future evidence-based structural modifications in order to obtain compounds more efficient towards *T. gondii* and less toxic for the host.

## 5. Conclusions

The synthesis and evaluation of activity of AADs with enhanced anti-*T. gondii* effect and diminished cell toxicity, along with providing the necessary infrastructure and know-how for the continuous synthesis and structural modifications of potential drug candidates, based on experimental results, is a long-term pursuit. Structural modifications of synthesized AADs should be performed carefully upon results of in vitro experiments, but also of in vivo experiments to develop compounds potent enough to prevent relapses and enable eradication of the parasite from the infected host, without causing toxicity. Three of the tested AADs (AAD1, AAD2 and AAD8) could potentially be included in future in vivo experiments, since they displayed the most potent in vitro anti-*T. gondii* activity in this group and possess satisfactory solubility and stability for in vivo studies. Considering the results presented in this study, our previous findings on acridine ring-DNA interactions, as well as our literature overview, the design of new AADs as potent anti-*T. gondii* agents could be based on the introduction of electron-donating substituents in the acridine ring of AAD1, AAD2 and AAD8, as well as on the introduction of 6-alkoxy-2-naphthyl, 4-piperidinylmethylene and hydroxamate-based pharmacophores in the amino acid side chain. Each compound of acceptable toxicity and activity on tachyzoites should be tested on *T. gondii* bradyzoites, to provide drug candidates competitive to standard treatment options.

## Figures and Tables

**Table 1:** Cytotoxic activity of evaluated AADs on non-infected Vero cells.

Compound (AAD)	CC_50_ VALUE (µM)
AAD1	41.72
AAD2	60.22
AAD3	88.37
AAD4	133.70
AAD5	54.66
AAD6	154.10
AAD7	92.95
AAD8	102.00
AAD9	50.19
AAD10	176.80
PYR+SDZ	2056.00

AAD—N-(9-acrydinil) amino acid derivative; PYR+SDZ—pyrimethamine and sulfadiazine combination treatment.

**Table 2:** Anti-*T. gondii* activity of the evaluated AADs.

Sample	Viability Reduction of *T. gondii* Tachyzoites (%)
AAD1	24.7
AAD2	25.8
AAD3	15.2
AAD4	4.5
AAD5	0.0
AAD6	22.0
AAD7	20.5
AAD8	33.3
AAD9	21.9
AAD10	21.9
PYR+SDZ	35.6

AAD—N-(9-acrydinil) amino acid derivative; PYR+SDZ—pyrimethamine and sulfadiazine combination treatment.

**Table 3:** Docking scores of tested AADs.

	7Q4A	2O2S	5E5I	8BIS	4WG3	4KYA	5XIG
Co-crystallized ligand	−13.95	−10.77	−10.16	−5.75	−13.60	−15.21	−10.54
AAD1	−14.34	−11.92	−7.91	−3.27	−12.19	−10.64	−9.37
AAD2	−13.61	−12.68	−9.62	−1.88	−10.86	−11.88	−10.57
AAD3	−14.13	−10.20	−7.86	−1.83	−12.76	−11.15	−8.69
AAD4	−10.58	−12.04	−10.77	−6.24	−11.69	−12.26	−9.25
AAD5	−14.07	−14.74	−8.86	−1.61	−11.50	−11.76	−9.36
AAD6	−12.91	−11.52	−8.75	−3.10	−11.59	−12.00	−9.09
AAD7	−12.67	−12.67	−8.77	−6.21	−11.99	−10.67	−10.08
AAD8	−12.18	−11.80	−8.14	−7.03	−11.11	−10.40	−10.05
AAD9	−11.94	−13.28	−7.54	−3.87	−11.15	−11.76	−8.72
AAD10	−10.26	−11.48	−7.56	−5.38	−11.26	−10.12	−9.84

AAD—N-(9-acrydinil) amino acid derivative.

## Data Availability

The raw data supporting the conclusions of this article will be made available by the authors on request.
